# Understanding chemically processed solar cells based on quantum dots

**DOI:** 10.1080/14686996.2017.1317219

**Published:** 2017-05-15

**Authors:** Victor Malgras, Andrew Nattestad, Jung Ho Kim, Shi Xue Dou, Yusuke Yamauchi

**Affiliations:** ^a^ International Center for Materials Nanoarchitectonics (MANA), National Institute for Materials Science (NIMS), Tsukuba, Japan; ^b^ Intelligent Polymer Research Institute, University of Wollongong, North Wollongong, Australia; ^c^ Institute for Superconducting and Electronic Materials, University of Wollongong, North Wollongong, Australia

**Keywords:** Solar cells, photovoltaics, quantum dots, lead sulfide, heterojunction, 60 New topics/Others, 101 Self-assembly / Self-organized materials, 103 Composites, 201 Electronics / Semiconductor / TCOs, 204 Optics / Optical applications

## Abstract

Photovoltaic energy conversion is one of the best alternatives to fossil fuel combustion. Petroleum resources are now close to depletion and their combustion is known to be responsible for the release of a considerable amount of greenhouse gases and carcinogenic airborne particles. Novel third-generation solar cells include a vast range of device designs and materials aiming to overcome the factors limiting the current technologies. Among them, quantum dot-based devices showed promising potential both as sensitizers and as colloidal nanoparticle films. A good example is the p-type PbS colloidal quantum dots (CQDs) forming a heterojunction with a n-type wide-band-gap semiconductor such as TiO_2_ or ZnO. The confinement in these nanostructures is also expected to result in marginal mechanisms, such as the collection of hot carriers and generation of multiple excitons, which would increase the theoretical conversion efficiency limit. Ultimately, this technology could also lead to the assembly of a tandem-type cell with CQD films absorbing in different regions of the solar spectrum.

## Graphical abstract

**Figure:**
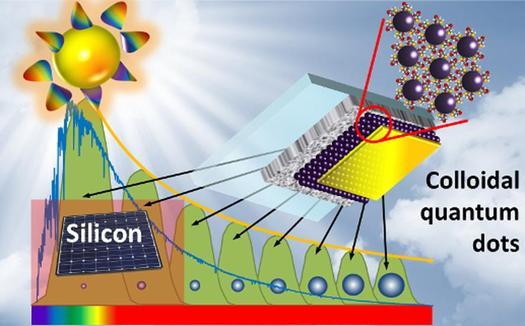

## 1. Historical aspects

A.E. Becquerel observed the photovoltaic effect for the first time in 1839 by detecting small currents when silver chloride was illuminated [1]; but it was only in 1883 when C. Fritts deposited selenium on a thin layer of gold that the junction solar cell was first produced, albeit with an efficiency below 1%. The early 20th century is marked by significant advances in crystallography (P. Curie), solid state physics (J.J. Thomson, P. Drude, P. Debye, F. Bloch) and statistical physics (A. Einstein, M. Planck, L. Boltzmann), which provided the necessary knowledge to understand semiconductor-junction-based photovoltaic devices. Various architectures were attempted before D. Chapin developed a doped (diffused) silicon *p*-*n* junction based solar cell in Bell Laboratories in 1954 following R. Ohl’s patent. The device, with an efficiency of around 6%, announced the first generation of commercially relevant solar cells. Most contemporary solar panels are still built on this crystalline silicon *p*-*n* junction technology attaining an efficiency of 26.3% (commercially available 21.5%) [2]. Combined with the invention of the transistor in 1947 (J. Bardeen, W. Shockley, and W. Brattain), which replaced *vacuum tube technology* by scalable electronics, the demand for manufactured semiconductors increased significantly. The price of silicon based solar cells dropped from USD 76.67/watt in 1977 to USD 0.60/watt in 2015, making the sun a competitive energy source, substituting for coal and other fossil fuels [3]. Nevertheless, researchers are still aiming to improve stability (life span, heat/moisture resistance), recyclability and especially conversion efficiency and fabrication costs.

**Figure 1.**
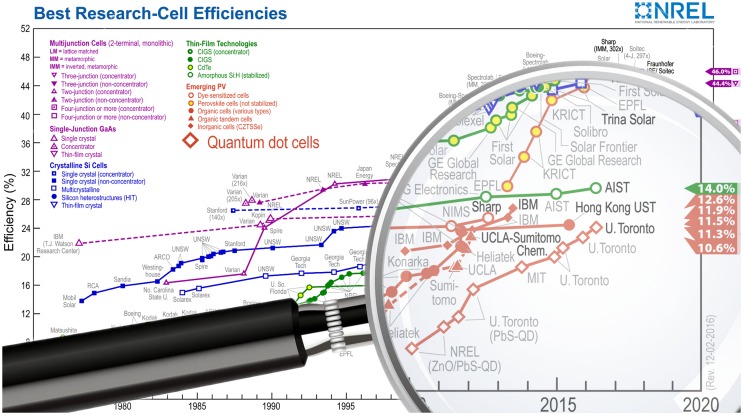
Best Research-Cell Efficiencies, adapted with permission by the National Renewable Energy Laboratory [6].

For multiple reasons, researchers had to look in other directions, as this technology started to reveal certain limitations. W. Shockley and H. Queisser calculated in 1961 a theoretical limit specific to this type of single junction in *bulk* semiconductor solar cells restricting the efficiency to 33.7% (for 1 sun illumination) [4]. Moreover, typical silicon purification lines require 650°C baking processes [5], which are responsible for most of the energy cost of production. The National Renewable Energy Laboratory (NREL) keeps a detailed track of the certified efficiencies of various photovoltaic technologies which have appeared since 1975 (Figure 1). The second generation of solar cells was aimed towards ecologically sustainable solutions and tried to decrease the amount of matter involved in the architecture of the device by using strongly light-absorbing materials such as 2–4 μm copper-indium-gallium-selenide (CIGS) thin films, which efficiently harvest most of the light in the 400–800 nm range. This technology can now achieve 21.7% conversion efficiency [7]. The second generation also includes organic and dye-sensitized solar cells which are assembled through relatively simple and low-cost processes and are able to reach efficiencies close to 12% [2]. The latter attracted considerable attention because of their *do it yourself* potential (simple technological manufacturing and low material purity requirements). These devices suffer from relatively short life-spans and instability, due to the use of molecular absorbers and liquid electrolytes, which make the devices hard to encapsulate. More recent research tends to address this drawback by using solid-state hole transporting materials [8], ionic liquids [9], or photonic crystal [10].

The third generation solar cells target various strategies to overcome the Shockley–Queisser limit. The present record comes from tandem cells with 46% efficiency (using a concentrator), resulting from the stacking of several *p*-*n* junctions made from elements optimized to absorb specific regions of the solar spectrum. Unfortunately, such technology requires metalorganic vapour phase deposition techniques, which increase the cost of production by several orders of magnitude, thus making it only suitable for aerospace applications.

Another approach consists of using quantum dots (QDs) as light absorbers. Under a specific size, certain binary crystals show significant changes in their optoelectronic behaviour, making them an attractive option for photovoltaic technologies. The interest for quantum dot-based solar cells started when A. J. Nozik assumed in 2001 that marginal phenomena such as hot carrier collection and multiple exciton generation could significantly improve solar cell performances, and thus overcome the Shockley–Queisser limit [11,12]. Different methods exist to synthesize these nanocrystals, such as vapour-liquid-solid, molecular beam epitaxy, electron beam lithography, successive ionic layer adsorption and reaction, and the synthesis of colloidal quantum dots (CQDs) through nucleation processes.

The former three are *physical syntheses* and require highly controlled atmosphere, high voltage, and/or high vacuum, which hinder their widespread application. The other methods, known as *chemical syntheses*, are relatively cheap to set up, but require significant optimization in order to obtain controlled size and size distribution. Moreover, one has to replace the long organic ligand used for the synthesis process, capping the colloidal QDs to prevent agglomeration, by smaller molecules. There is a great deal of research which is currently aiming to improve this *ligand exchange* method and thus improve the performance and stability of the device. There are three main designs that have been investigated to achieve proper photovoltaic devices: the Schottky junction, the quantum dot sensitizer and the depleted heterojunction. The last architecture has recently achieved 10.7% efficiency through the use of hybrid passivation methods [13]. This review presents a brief survey of the typical principles of operation of solar cells, and then focuses on colloidal quantum dot-based devices in their technological context.

## 2. Operation of solar cells

Solar cells can be seen as diodes in which the generation current can be greatly increased due to the ability of the material to absorb photons, thus exciting electrons which will contribute to the typical thermally generated current.

### 2.1. Solar spectrum and solar simulator

**Figure 2.**
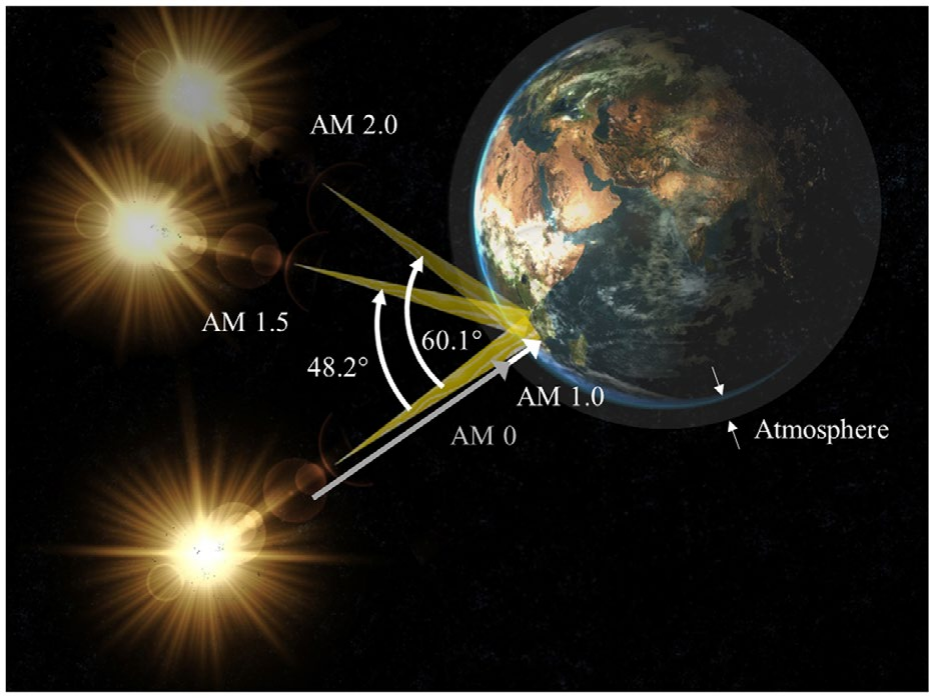
Geometric representations of the various solar spectrum standards AM 0, AM 1.0, AM1.5 and AM 2.0.

Many factors can affect spectral irradiance distribution, such as the latitude, time of the year, and time of the day, as well as the weather conditions, e.g. clouds, humidity, and wind. In order to define a *standard sun* used to compare the efficiency of photovoltaic devices, one can refer to the air-mass (AM) index that relates to different conditions. AM 0 corresponds to the solar spectrum above before reaching the atmosphere and AM 1.0, 1.5, and 2.0 express the solar irradiance from the sun after passing through the atmosphere with angles of 0, 48.2 and 60.1°, respectively (see Figure 2). This gaseous mass is composed of various compounds which absorb a significant proportion of the light intensity (up to 23%). AM 0 is therefore only suitable for extraterrestrial applications (e.g. satellites) while the others provide insight on the input power a solar cell can absorb in a day. AM 1.0 is exact only for devices installed in equatorial or tropical regions at the zenith. Most of the Earth’s population live further from the equator in temperate zones where the light path across the atmosphere is longer, so AM 1.5 represent a much more relevant standard. Some other factors include the *albedo* of the surroundings (diffuse reflectivity of a surface). For these reasons, most solar simulators use a xenon arc lamp with appropriate filters mimicking the AM 1.5 spectrum.

### 2.2. p-n junction under illumination and the Shockley–Queisser limit

Under dark conditions, the generated current comes from thermally activated charge carriers. Photons conveying more energy than phonons (lattice vibrations, *E*_*ph*_ < 100 meV), their contribution to the generation current can quickly become significant. Indeed, most of the solar spectrum is spread between 250 nm (4.96 eV) and 4000 nm (0.31 eV), divided between ultraviolet (UV), visible, and infrared (IR) light.

**Figure 3.**
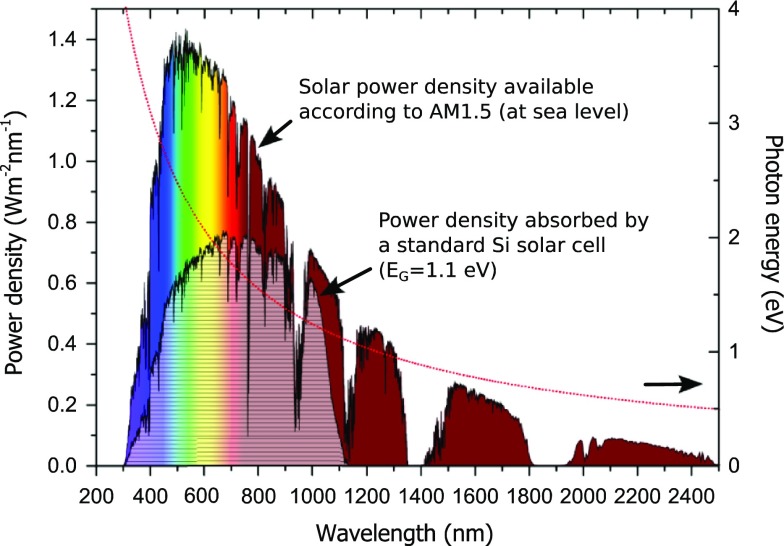
AM 1.5 (blue) solar power and proportion which is actually absorbed by a standard crystalline silicon solar cell (purple). The orange dashed line represents the energy carried per photon at a specific wavelength.

As described by Shockley and Queisser in 1961 [4], the mechanisms responsible for conversion and extraction limit the efficiency of standard solar cell to 33.7%. First, photons with energy lower than the forbidden bandgap of the material (*E*_*G*_) will be diffracted, reflected, or transmitted through the junction. This phenomenon is accountable for the loss of 19% of the solar energy in a typical standard crystalline silicon solar cell with 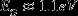 (see Figure 3). Secondly, in the case where a photon transfers an energy *E*_*hν*_ higher than *E*_*G*_ to an electron, the latter will be excited to a higher energy level to further thermalize to the bottom of the conduction band (*E*_*CB*_) by releasing a phonon with an energy *E*_*ph*,*e*_ (analogously *E*_*ph*,*h*_ for holes) with *E*_*ph*,*e*_ + *E*_*ph*,*h*_ = *E*_*hν*_ – *E*_*G*_. This other mechanism is responsible for 33% solar power loss. Finally, phenomena such as the radiation of the photovoltaic device (black body radiation) and radiative recombination (detailed balance principle) also account for another ~15% loss of the incoming solar energy.

**Figure 4.**
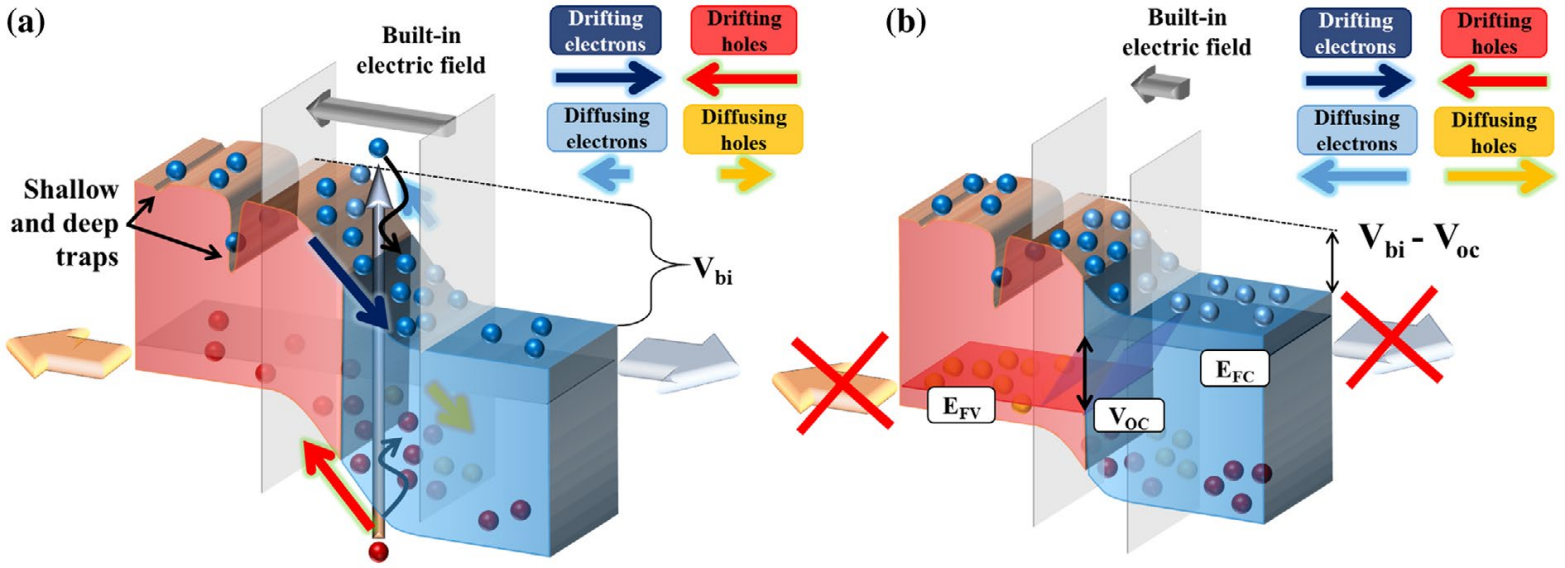
Illustration of the band diagrams at a p-n junction under short-circuit (a) and open-circuit (b) conditions. All terms are defined in the text.

In a solar cell under *short circuit conditions* (Figure 4(a)), the diffusion flow remains unchanged, but most of the photogenerated charges drift along the electric field. The short-circuit current density, *J*_*sc*_, is maximum and corresponds to the photogenerated charges diffusing towards the depletion region to be driven along by the junction polarity *V*_*bi*_ (the built-in potential). The main limitation resides in properties such as the diffusion length and the minority carriers’ lifetime, which can bring them to recombine before reaching the electric field. If a load resistance is added to the circuit, however, charge extraction is restricted. Once the collection rate decreases below the photogeneration rate, excess minority carriers accumulate on each side of the depletion region, gradually splitting the quasi-Fermi levels associated to the valence and conduction bands (*E*_*FV*_ and *E*_*FC*_, respectively) and building up a polarity opposed to the applied potential drop. This causes the diffusion current to increase. The recombination probability (or recombination rate) depends strongly on the number of excess carriers, until equilibrium conditions are reached to satisfy:(1)
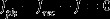



with *J*_*rec*_ being the overall recombination current density, *J*_*ph*_ the photogenerated current density and *J* the current density exiting the cell. Any photogenerated charges which cannot be extracted will thus necessarily recombine.

Under *open circuit conditions* (infinite load resistance), excess charges are confined in the device, and equilibrium is reached when the generation and recombination rates are equal (Figure 4(b)). Under these conditions, each side of the depletion region hosts its maximum possible carrier density, and the quasi-Fermi levels are separated by an energy *qV*_*oc*_, where *V*_*oc*_, the open-circuit voltage, represents the maximum electrical potential which can be achieved in the device.

Any intermediate states of the charge flow can theoretically be derived from Shockley ideal diode approximation:(2)
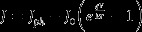



This typical current density–voltage relation is the principal figure of merit to assess the performance of a solar cell and is further discussed in the following section.

### 2.3. Solar cells characteristics: ideal vs. real

The principal information regarding the performance of a solar cell resides in the current density – voltage (*J* – *V*) characteristic. The short-circuit current density (*J*_*sc*_) is the maximum current that can be collected from the device and reflects the output of a broad set of properties such as photo-absorption, injection/diffusion, junction engineering, and defect/impurity levels. In general, *J*_*sc*_ will depend on:

• the ability of the active material to strongly absorb light;
• how fast is the injection from the absorbing material to the transport material compared to the back-surface recombination process; and
• the potential distribution through the cell containing the least barriers/well which could act as recombination centres.

For optimally engineered solar cells, the short-circuit current density can be expressed as:(3)
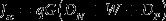



where 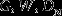 and *D*_*P*_ are the charge generation rate (includes absorption spectrum and injection rate), the depletion region’s width, and the diffusion lengths of minority carriers (electrons and holes), respectively.

**Figure 5.**
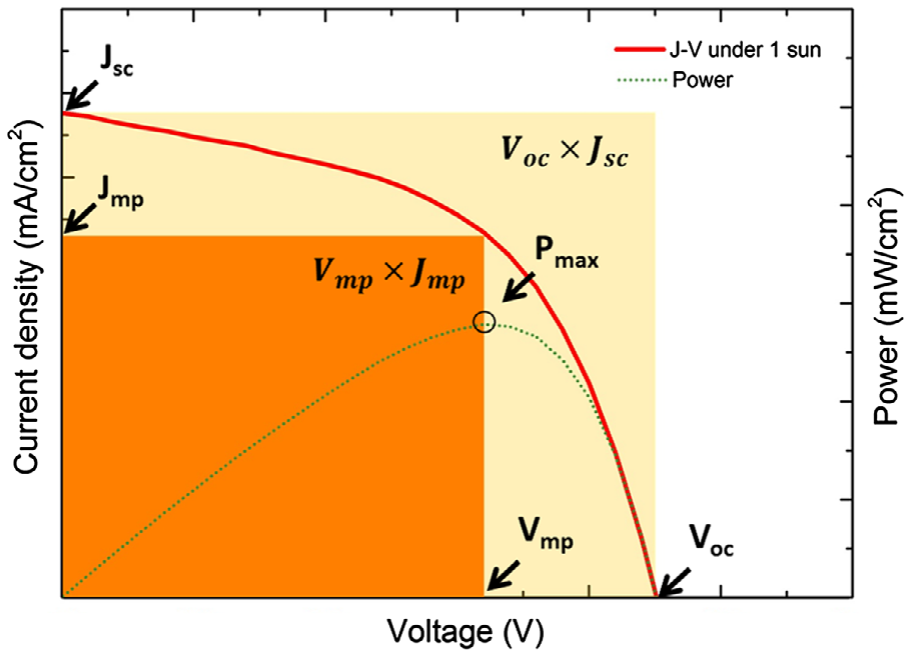
A typical J – V curve and main parameters as defined in the text.

Figure 5 shows a typical *J* – *V* curve and highlights the most relevant parameters. The maximum power, *P*_max_ with coordinates (*V*_*mp*_, *J*_*mp*_), which can be obtained by plotting the power curve *P* = *V* × *J*, determines under which regime the solar cell should operate in order to optimize its output. From this value, one can calculate the *fill factor* (FF):(4)
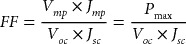



This ratio provides insight on the *squareness* of the solar cell response. Higher fill factors are attributed to more ideal devices: they result in higher and more stable power as the input conditions fluctuate. Finally, the device efficiency represents the ratio between the maximum power output and the power input:(5)
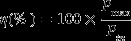



with 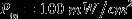 for the 1.5 AM standard.

## 3. Quantum dots: properties and synthesis

Quantum dots are small particles (or nanocrystals) with electronic properties which differ from those of their bulk counterpart due to their reduced size. This section compiles their properties of interest in the context of solar cells, the different methods of synthesis, and their role in different photovoltaic device architectures.

### 3.1. Confinement in quantum dots

The so-called *particle*-*in*-*a*-*box* model is the most comprehensive example to introduce confinement in quantum mechanics, describing how the energetic configuration of a particle depends on the size and shape of the space it is confined in. The energy levels available for a particle of mass *m*, confined in a box of size *L*, can be obtained by solving the Schrödinger equation for the single dimension case with infinite potential boundaries [14]:(6)
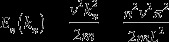



where *ν*, *k*_*n*_ and *n* are respectively the reduced Planck constant, the wavenumber and an integer justifying mathematically the terms *discrete* (or *quantized*) energy levels. The energy between *E*_*n*_ and *E*_*n*+1_ increases as the size of the box *L* decreases and becomes negligible at macroscopic levels.

QDs are semiconducting nanocrystals that are small enough to be considered as potential wells (similar to the particle-in-a-box) within which electrons undergo confinement regime. This regime is considered to be *strong* when the size of this three-dimensional box becomes smaller than the theoretical distance between embedded electron and hole, the exciton Bohr radius *a*_*exc*_:(7)
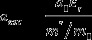



with 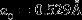, *ɛ*_*r*_, 
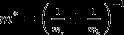
, and *m*_0_ being the hydrogen atom’s Bohr radius, the relative permittivity constant of the material, the electron-hole reduced mass, and the free electron mass, respectively. For example, in the case of lead sulfide with *m*_*e*_ = *m*_*h*_ = 0.08*m*_0_ and *ɛ*_*r*_ = 17.2, we obtain 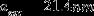. This value is a first approximation, as it does not take into consideration effects due to the dielectric properties of the crystal. Other factors will also be responsible for modifying the boundary potential seen by the confined charge in a nanocrystal, and thus affecting the energy states distribution, such as shape symmetries (or asymmetries), surface reconstruction and additional chemical interactions.

### 3.2. Tunable bandgap

**Figure 6.**
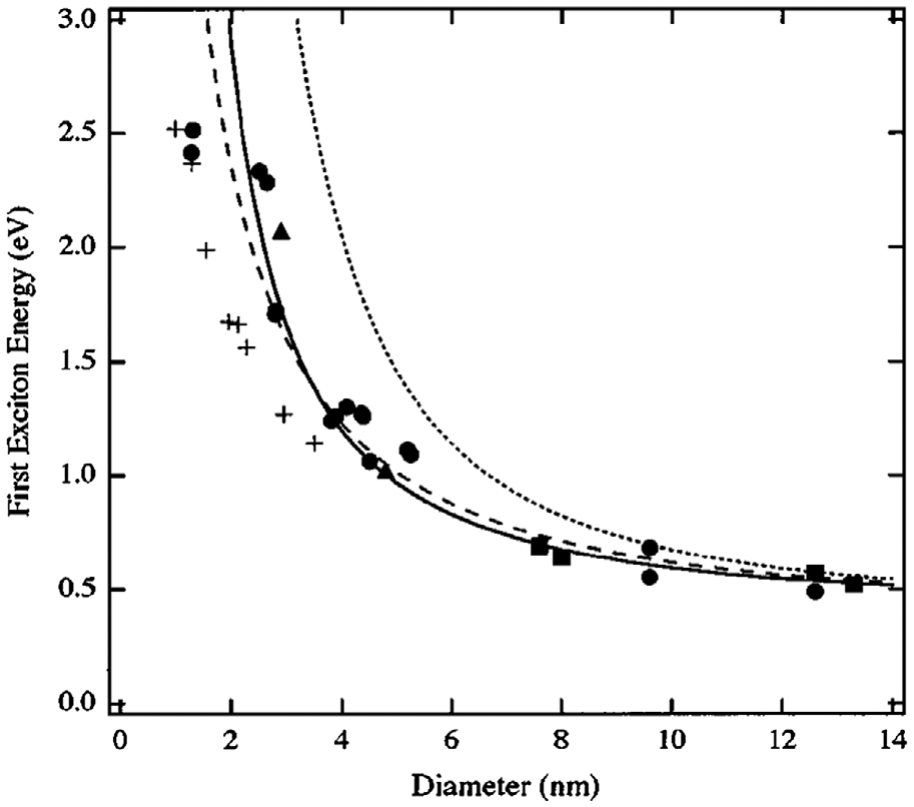
First excitonic energy dependence on the crystal diameter of the effective mass model (dotted curve), the hyperbolic model (dashed curve), and the four-band model (solid curve). Symbols are experimental data from various publications. Reprinted with permission from [17]. Copyright 1997 Optical Society of America.

In the quantum confinement regime, variations in band edge energy level positions will become significant. Louis E. Brus [15] first reported the effective mass model to evaluate the bandgap of QDs:(8)
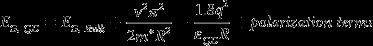



where *R* and *ɛ*_*QD*_ are the radius and the dielectric constant of the QD, respectively. Lead chalcogenides, however, have relatively high dielectric constants and small bandgaps (see Table 1). The model deviates from real experimental data for crystal sizes under 10 nm (see Figure 6) as the common approximations employed to solve the Schrödinger equation do not hold anymore. Wang et al*.* [16] developed a hyperbolic model overcoming this discrepancy and rewrote the equation as:(9)
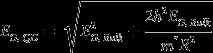



**Table 1.** Dielectric constants (*ɛ*_∞_), bandgaps **(E**_**G**_ **)** and reduced masses for electron (*m*_*e*_/*m*_0_) and holes (*m*_*h*_/*m*_0_) of lead chalcogenides PbS, PbSe and PbTe.

		PbS	PbSe	PbTe
*ɛ*_∞_		17	23	33
*E*_*G*_ (eV)		0.37	0.27	0.32
*m*_*e*_/*m*_0_		0.080	0.040	0.024
*m*_*h*_/*m*_0_		0.075	0.034	0.022

Another method was later proposed, using a complex four-band model [17,18] using the 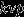 Hamiltonian:(10)
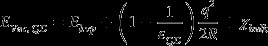



where 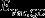 is the energy level relative to vacuum, *E*_*kνp*_ is the energy level from the 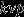 calculation, and *χ*_*bulk*_ is the electron affinity of the bulk.

When it comes to band structure engineering of electronic devices and specifically designing the desired properties of homo- or heterojunctions, tuning the bandgap has significant advantages. For example, many photovoltaic devices require the use of type II semiconductor junctions (staggered gap) for effective charge injection, transport, and collection.

### 3.3. Electron–hole pairs, excitons

In bulk junction semiconductor devices, *electron*–*hole pairs* are formed by considering the final state of an excited electron as being *in* the conduction band, leaving a hole behind in the valence band and having both charges swept away from each other by the electric field in the depleted region. In reality, however, this only holds for macroscopic materials where the long-range periodicity of the lattice ensures that the electronic properties remain locally the same, wherever the charges are located. In nanostructured devices, many new parameters must be taken into account: crystal boundaries, shape effect, and interface tunnelling. They can be seen as defects introducing perturbations which result in potential wells, barriers and mid-gap states.

**Figure 7.**
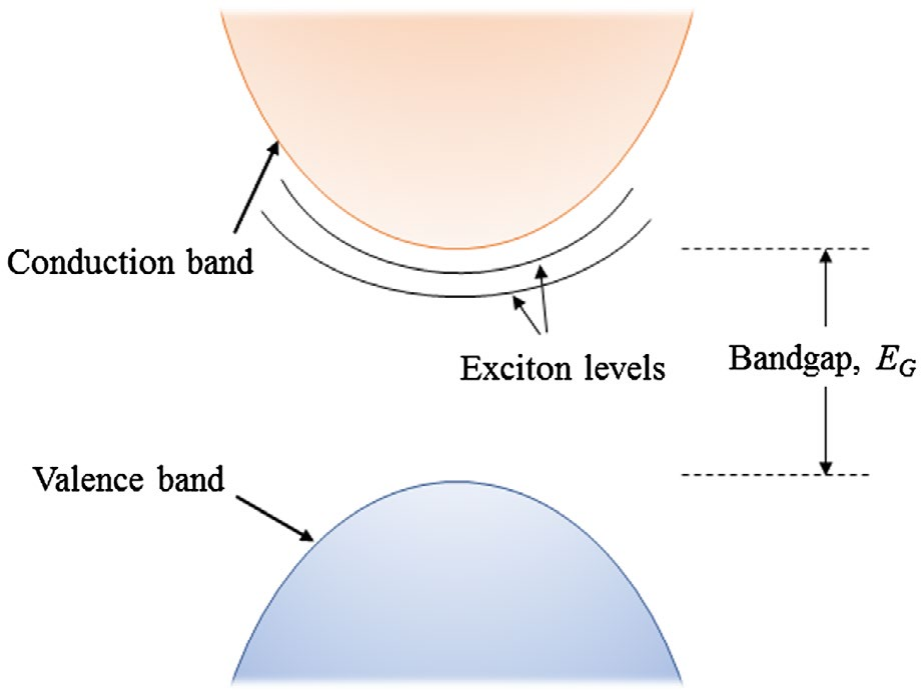
Schematic representation of excitonic levels located within the bandgap.

For example, in crystals with high dielectric constants, the Coulomb interactions between electrons and holes can be screened. Therefore, they become weakly bound and form a quasi-particle called an *exciton*. Its energy state can be calculated by solving the Schrödinger equation for the hydrogen atom, replacing the mass of the proton by the effective mass of the hole from the material considered. In bulk materials, the excitonic levels are located in the bandgap near the conduction band (see Figure 7), reflecting the Coulomb interaction. Excitons have no net charge, but can travel in a medium until they receive enough energy to split. In bulk materials, this can be as low as the thermal vibrations at room temperature (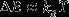). In isolated quantum dots (such as PbS quantum dots), it is well established that there is no *free charge* state, because the excited electrons and holes are spatially confined, and the whole transitional spectrum is entirely dictated by excitonic levels [19–23].

### 3.4. Relaxation dynamics, hot carriers, and multiple exciton generation

When an electron absorbs a photon with an energy which is above the energy of the lowest exciton energy (generally referred as 1S_h_-1S_e_), various pathways can occur:

(1) the electron may thermalize to its lowest state by dissipating energy as lattice vibration through electron-phonon interaction or Auger process;
(2) the excess energy can be transferred to one or more electrons through reverse Auger process, resulting to multiple exciton generation (MEG); and
(3) the electron–hole quasi-particle can split, leaving a highly energetic charge (hot carrier) that must be extracted rapidly before recombining.

Pathway (1) results in an obvious energy loss (Shockley–Queisser limit) and is still the most probable fate of excited charges because of its extremely fast occurrence in bulk semiconductors (~ps). Theoretical models have shown that these processes can be slowed down when the photogenerated carrier density is increased up to ~10^18^ cm^−3^, thus inducing a *hot phonon bottleneck* due the non-equilibrium distribution of longitudinal optical (LO) phonons (pathways (2) and (3)) [24,25]. Later on, Nozik and co-workers showcased the potential of this effect if applied in optoelectronic devices [26–28]. This process is, however, still limited by crystal momentum which must be conserved during the transitions. Therefore, MEG was only be observed in bulk semiconductors for *hν* > 4*E*_*G*_ [29]. For QD-based devices, however, research groups report more and more promising results demonstrating enhanced photoconversion efficiencies through MEG, even under AM 1.5 conditions [30–32].

**Figure 8.**
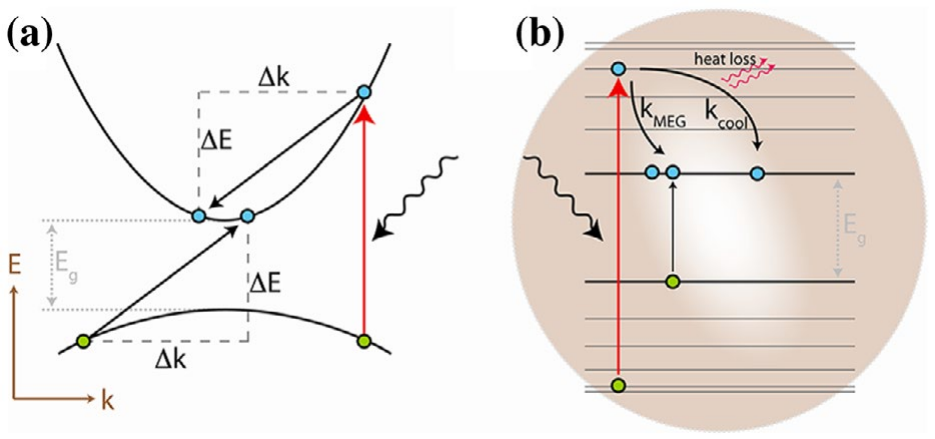
Fast relaxation in continuous energy levels (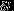) and conservation of crystal momentum (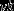) in (a) bulk semiconductors versus (b) MEG in nanocrystals. Reprinted with permission from [29]. Copyright 2013 American Chemical Society.

Because of their small size, QDs do not suffer from the limitations related to conservation of momentum which is generally inherent to long-range periodicity (Figure 8). These assumptions arise from the idea that if the energy separation between two discrete exciton levels is higher than the fundamental phonon energy, multiple-phonon processes would be necessary in order for the charge to relax to the lowest level. These mechanisms are significantly slower than single phonon interactions, and their relaxation time could then be estimated from:(11)
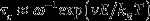



where *τ*_*c*_ is the hot carrier cooling time, *ω* is the phonon frequency and *νE* is the energy level separation. According to Equation (11), strongly quantized levels (>0.2 eV) would extend the relaxation time to ~100 ps. Using ultra-fast transient absorption spectroscopy or time-resolved photoluminescence decay, Schaller et al*.* observed different decaying components, which were associated to single and multiple excitons [33–35].

**Figure 9.**
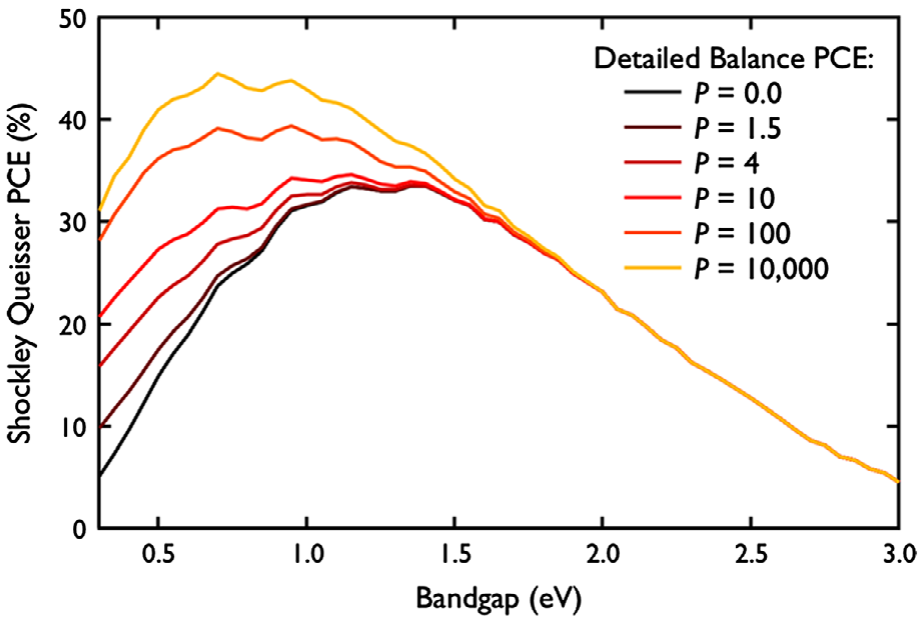
Theoretical improvement of the Shockley–Queisser limit due to the MEG efficiency P. PCE: photoconversion efficiency. Reprinted with permission from [29]. Copyright 2013 American Chemical Society.

These properties, if fully exploited in solar cells technology [12,29], are expected to enhance the Shockley–Queisser limit (Figure 9) from 33.7% to 45% (for MEG) [36] and to 67% (for hot carriers collection) [37]. Until now, however, these measurements were only successfully performed on individual nanocrystals under controlled conditions. The challenge of incorporating QDs into a photovoltaic device while taking advantage from MEG [38–41] or hot carrier [42,43] mechanisms is still attracting a lot of attention.

### 3.5. Synthesis

Different methods have been investigated to produce QDs of different materials, with various shapes, sizes and size distributions for a multitude of applications. Physical-chemical vapour deposition techniques generally involve the growth/formation of the materials directly on a substrate, giving an improved control over the size and spatial distribution. They are especially appropriate for the fabrication of superlattices which amplify quantum electronic confinement properties [44–46]. On the other hand, wet chemical techniques provide good alternative routes to producing QDs in a colloidal suspension (CQDs) with 3D quantum confinement characteristic. These methods typically use standard glassware with temperatures below 300 °C, significantly reducing the production costs.

#### 3.5.1. Physical vapour deposition

**Figure 10.**
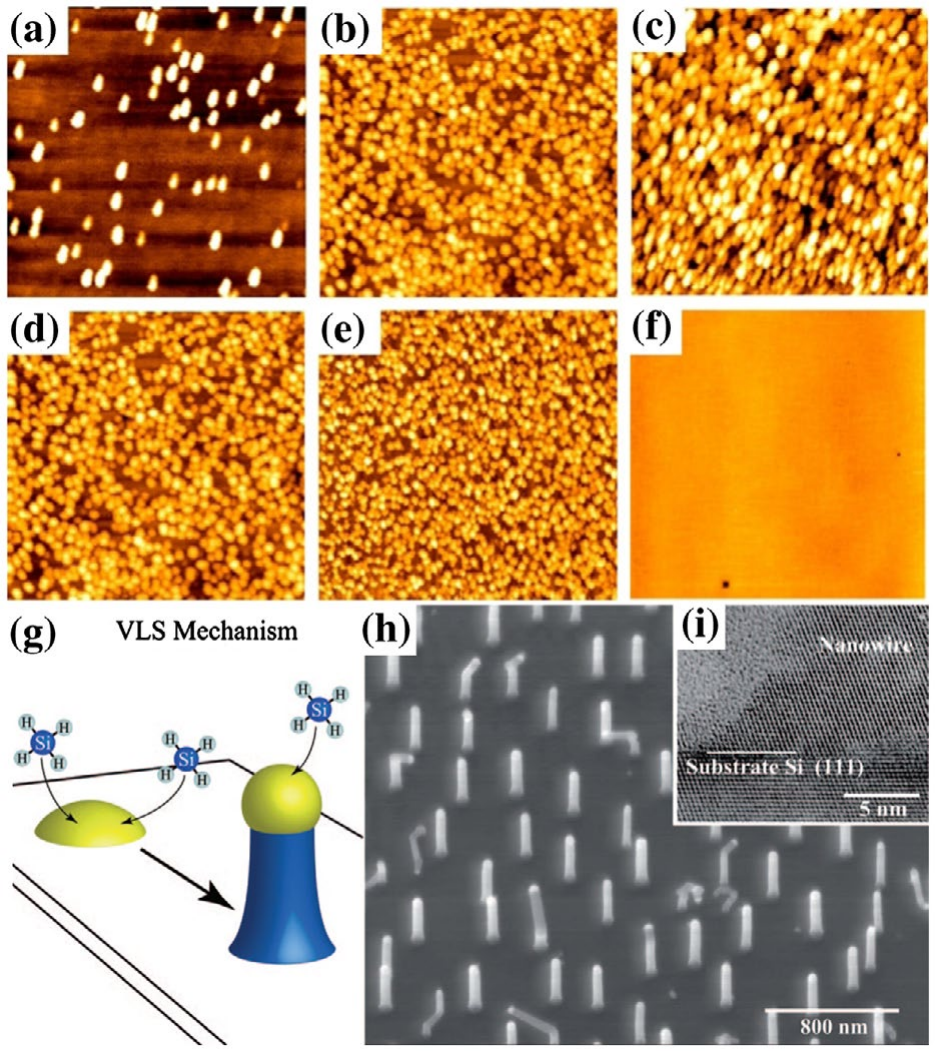
InAs islands grown at different V/III ratios [(a) 15, (b) 25, (c) 35] and different temperatures [(d) 400°C, (e) 450°C, (f) 500°C]. Reprinted with permission from [50]. Copyright 2013 Institute of Physics. (g) Schematic of VLS mechanism. Reprinted with permission from [51]. Copyright 2010 American Chemical Society. Low (h) and high resolution (i) transmission electron microscopy images of Si nanowires. Reprinted with permission from [52]. Copyright 2005 Hanser.

A standard method to grow a 3D structure through vapour phase deposition is the Stranski–Krastanov growth [47–49]. By depositing several monolayers of semiconductors with a strong lattice mismatch, epitaxial growth can be initiated in a layer-by-layer fashion and to coherently grow 3D islands (Figure 10(a)–(f)) [50].

Another widely reported technique is the vapour-liquid-solid (VLS) deposition [51–55], where a thin film of gold (1–10 nm) is typically deposited onto a silicon wafer (100) and heated above the Au/Si eutectic point to form droplets of Au–Si alloy on the surface of the substrate. The sample is then placed inside a vacuum chamber with a flow of reactive gas mixture (typically SiCl_4_:H_2_) at 800°C. The gas is absorbed into the droplets, which act as a catalyst to lower the activation energy of normal vapour solid growth, until supersaturation is reached. This is followed by the excess Si atoms to be automatically driven down to the substrate, leading to anisotropic growth (see Figure 10(g)–(i)).

#### 3.5.2. Successive ionic layer adsorption and reaction (SILAR)

Vogel et al*.* [56] reported the sensitization of wide-band-gap semiconductors with various binary sulfides semiconductor nanoparticles through chemical bath deposition. The method was further renamed later on as SILAR in order to prevent the confusion with other types of chemical bath deposition techniques. The technique consists in the successive immersion of the substrate in aqueous solution of salts (e.g. lead nitrate followed by sodium sulfide). The deposition can be controlled by varying the immersion time, the number of repetitions, the type of salt or the concentration. The number of *seeds* deposited during the first cycle remains a limiting factor, however, as any subsequent steps will only *feed* the pre-existing crystallites. The direct growth on the substrate has the advantages of increasing the cohesion of the sensitizer and thus improving electron injection. This method, which, for instance, is used for the fabrication of quantum dot sensitized solar cells, suffers still from certain drawbacks which will be discussed in section 4.1.1 [57].

#### 3.5.3. Colloidal growth synthesis

**Figure 11.**
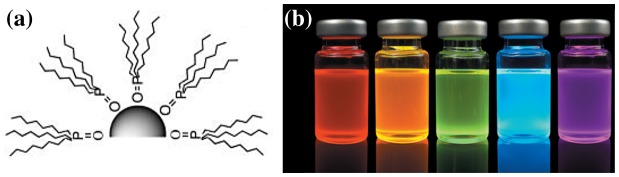
(a) QDs capped with tri-n-octylphosphine oxide. Reprinted with permission from [65]. Copyright 2005 Royal Society of Chemistry. (b) ZnCdSeS quantum dots with various sizes emitting at various wavelength. Reprinted with permission from [67]. Copyright 2016 Nature Publishing Group.

After Faraday synthesized gold colloidal nanoparticles in 1857 [58], many other chemical routes were developed in order to obtain similar nanostructures with a wide range of binary compositions. A typical method involves the combination of two (or more) precursors, generally from groups II/VI or IV/VI, in a hot solvent containing carefully selected coordinating molecules under vigorous stirring. At the start, a multitude of nucleation centres are formed, initiating the growth of particles through Ostwald ripening. The role of the coordinating ligands is to set a critical crystal size, after which the sterically hindered growth leads to a narrowed size distribution [59–61]. The mean size can be empirically controlled through parameters such as the precursor ratio, ligand concentration, temperature and reaction time. These techniques developed and modernized by Murray et al*.* [62–64] in IBM’s laboratory remain, even now, the standard recipes for the synthesis of cadmium and lead chalcogenides. Typically, tri-n-octylphosphine (TOP) is used to dissolve the chalcogen (S, Se, Te) precursor, and tri-n-octylphosphine oxide (TOPO) acts as the coordinating ligand (Figure 11(a)) [65]. They also introduced the combination of lead oleate-bis(trimethylsilyl)sulfide precursor to produce PbS quantum dots in hot diphenyl ether (boiling point ~260°C). Currently, researchers are generally adopting the Hines and Scholes method [66], in which toxic diphenyl ether is replaced by 1-octadecene (boiling point 315°C). The synthesis must be followed by appropriate washing to extract the quantum dots from the reaction solution and remove unreacted ligands and precursors. The final product remains capped with oleate (or TOPO) molecules, making it stable in non-polar solvents, hence the name *colloidal* quantum dots. Parameters such as injection temperature and reaction time can be accurately controlled to produce a wide range of nanoparticle sizes, and thus a wide range of bandgaps with different absorption cut-off and emission wavelengths (Figure 11(b)).

## 4. Quantum dots for photovoltaic application

QDs show unique optoelectronic properties due to their extreme confinement, including a high extinction coefficient allowing thin layers to absorb a significant portion of incident photons. There has been considerable research aiming to design devices with the purpose of optimizing photo-absorption and charge transport/collection while maintaining a high voltage output. Researchers use various architectures as scaffolds to observe the effects of new materials and new treatments, and to study the fundamentals of electronic transport.

### 4.1. Typical device architectures

**Figure 12.**
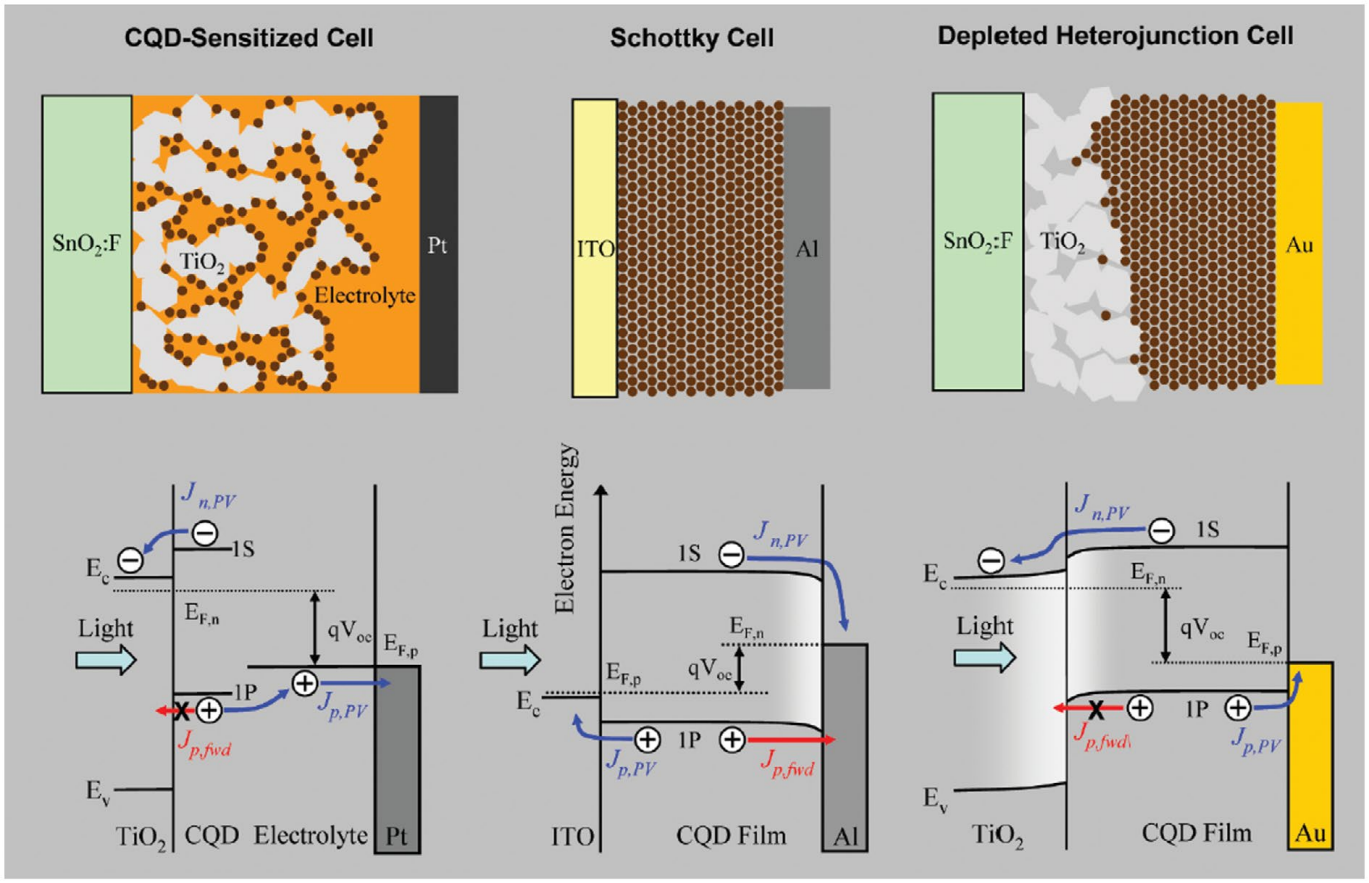
Schematic description of three quantum dot-based solar cells along with band diagrams illustrating the charge dynamic within the device. Reprinted with permission from [69]. ITO stands for indium tin oxide. Copyright 2011 American Chemical Society.

Here, three different architectures are reviewed (Figure 12): the quantum dot sensitized solar cell, the colloidal quantum dot Schottky junction solar cell and the colloidal quantum dot heterojunction solar cell [68,69]. Other strategies, not covered in the current review, have more recently been investigated, such as hybrid cells blending colloidal quantum dots with polymers [70–73], fullerenes [74,75] graphene [76,77], or carbon nanotubes [78].

#### 4.1.1. Quantum dot sensitized solar cell (QDSC)

Inspired by their organic counterparts (dye-sensitized solar cells, DSCs), the inorganic sensitizers from QDSCs are generally grown through SILAR deposition and are selected for being strongly light-absorbing in the visible range. The operation mechanism can be briefly summarized as: (i) a photon is absorbed in a QD, generating an exciton; (ii) the electron and hole dissociate at the interface with a TiO_2_ particle; (iii) the QD sensitizer is oxidized as the electron is injected into the TiO_2_ layer, and (iv) further transported towards the working electrode, which is typically a transparent conductive oxide (TCO); (v) the hole recombines with an electron from a redox medium and regenerates the ground state; and finally (vi) the system is at equilibrium once the oxidized electrolyte diffuses to the counter electrode where it is reduced.

Typical electrolytes in DSCs use the iodide/triiodide redox couple; it is however a reactive source of corrosion for the QD sensitizers. Other compositions, including polysulfides dissolved in methanol [79], cobalt complexes [80] or solid state hole conductors such as (2,2(,7,7(-tetrakis-(N,N-di-pmethoxyphenylamine) 9,9(-spirobifluorene) (spiro-OMeTAD) [81], have shown more stable performance. As in DSCs, the main detrimental pathway for photogenerated carriers to recombine in QDSCs is from the TiO_2_ conduction band into the redox couple from the electrolyte [82,83]. This can be explained from the low coverage efficiency during the SILAR deposition. Generally, a ZnS coating efficiently screens these back-recombination mechanisms, but it can also introduce new monoenergetic surface states affecting the fill factor [84]. Various other strategies are still being investigated [85–89]. The latest devices sensitized with PbS quantum dots showed short-circuit current as high as 20.8 mA cm^–2^, leading to an overall efficiency of 8.2% [90].

#### 4.1.2. CQD Schottky junction solar cell (SJSC)

The CQD SJSC was the first of its kind to achieve efficiencies beyond 1% from CQDs [91]. The architecture is based on overlaying a TCO with large work function (such as indium-doped tin oxide) with a film of *p*-type CQDs to form an Ohmic contact. This is followed by evaporating a metal with a shallow work function (aluminium, magnesium) to generate an appropriate band-bending suitable to extract electron while screening holes.

This attractive strategy had, however, a few limitations. The short diffusion length in these films limits their thickness to 200 nm, which is too thin to absorb a sufficient portion of the incident radiation. Increasing the thickness of the device above this critical limit causes charge recombination to become a substantial problem. Also, the Fermi level can be easily pinned by defect states at the metal/semiconductor interface, affecting the overall open-circuit voltage. Nevertheless, optimization of the material synthesis, post-treatments, and assembly [92], along with carefully engineered hole-selective contacts, allowed Piliego et al*.* [93] to produce devices with an efficiency of 5.2%.

#### 4.1.3. CQD depleted heterojunction solar cell (DHJSC)

This architecture has similar aspects to the CQD SJSC, except that it has an additional *n*-type layer of wide-band-gap semiconductor particles (TiO_2_, ZnO) between the TCO electrode and the CQD layer to secure electron transport. The back contact is typically made of a metal with a large work function (such as Au or Pt).

As compared to the SJSC, the mild depletion region of the heterojunction provides more efficient electron-hole dissociation, and because it is located on the illuminated side, carrier separation happens faster. Back electron transfers from the oxide to the CQD layer can be effectively suppressed by the built-in field. Finally, a higher open-circuit voltage can be achieved because of the large difference between the Fermi level of the TiO_2_ and the work function of the counter electrode. The first DHJSC [69] was reported in 2010 with an efficiency of 5.1%, far beyond the records previously achieved by other architectures at the time. This high performance was partly due to optimized parameters such as: CQD synthesis, size selection, ligand exchange and film thickness (both for TiO_2_ and PbS CQDs). Further improvement, including controlled oxide doping and inorganic passivation, enhanced the performance of these solar cells up to 7.4% [94–97]. Ultimately, strategies such as replacing the wide-band-gap oxide by n-doped CQD film and stacking films with different QD size were considered to fabricate promising tandem structures to increase the absorption range [98–101].

### 4.2. The role of the ligands

Ligands are ions or molecules coordinating with a metal atom [102]. In the context of nanocrystal chemistry, ligands form a bond with surface atoms where, by definition, the periodicity is interrupted and fulfils three main roles: passivation, functionalization, and steric spacing.

The term *passivation* literally implies that a material is made less reactive to its environment. The surface of nanoparticles can be unstable due to strains, uncontrolled reconstruction, or unbalanced charge. These reactive sites are ready to bond with any adventitious moieties so as to lower its surface energy. The most common contaminants are the oxides formed from oxygen and moisture in air. These species generally have a detrimental impact on the particle properties by adding new localized surface states (generally mid-gap states) to the overall crystal energy structure. These levels can not only pin the Fermi level down (and thus lower the open-circuit voltage), but also act as deep traps and recombination centres. Surface passivation usually involves the introduction of ligand molecules (or ions) to coordinate with these unstable sites. An efficient passivation will induce a minimum change in the energy state distribution, while preventing other adventitious contaminants from being adsorbed.

The term *functionalization* has a broad meaning, as it includes any modification in the physical or chemical reactivity of the material. For instance, in the context of biotechnology, CQDs can be functionalized to improve their biocompatibility [103] or can act as a fluorescent chromophore binding to specific cells or proteins [104]. For PbS CQD-based solar cells, the nanoparticles are coated with oleate ligands having long non-polar hydrocarbon chains. This has the effect of neutralizing the apparent surface charge, thus giving the material the ability to be suspended in non-polar solvents (e.g. alkanes, toluene, chloroform). This enable the ability of CQDs to be deposited on substrates through spin-coating, dip-coating or even potential screen- and inkjet-printing techniques [105].

**Figure 13.**
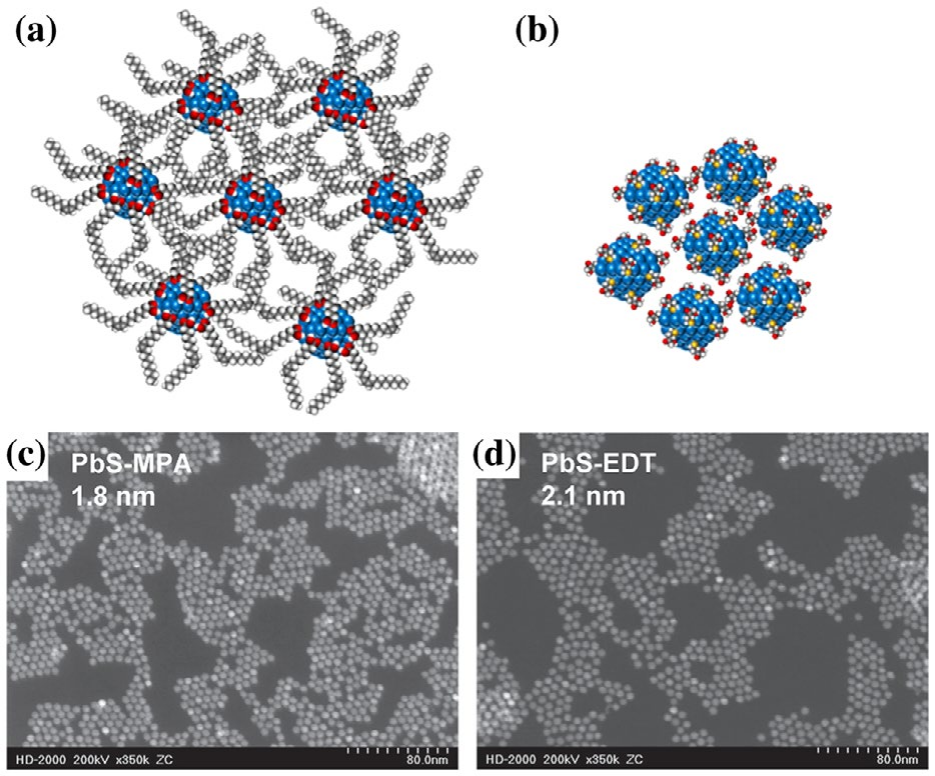
Representation of steric spacing between CQDs when using (a) oleic acid or (b) 3-mercaptopropionic acid. Colour codes are as follows: oxygen: red, carbon: grey, hydrogen: white and sulfur: yellow. Scanning transmission electron microscopy images of CQDs after MPA (c) and EDT (d) ligand exchange. Reprinted with permission from [115]. Copyright 2012 American Chemical Society.

In order for QDs to retain their confined optoelectronic properties, they must maintain a certain degree of isolation, thus preventing the electron wave function from delocalizing in neighbouring nanocrystals. The loss of confinement leads to uncontrolled and non-uniform energy level reconfiguration; the first exciton transition is reduced and the electronic landscape regains its continuous character (from the bulk). On the other hand, electrons from completely remote nanoparticles have a very low hopping probability and thus suffer from low conductivity. After being cast on a solid surface, ligands of different lengths provide various *steric spacing* between the QDs and a balance must be found between quantum confinement and electronic conductivity. For this reason, CQD films are generally made through a layer-by-layer process, where each cycle consists in exchanging long chain ligands for shorter ones (see Figure 13(a) and (b)). Ligand exchange can roughly be categorized into two groups: organic and inorganic.

#### 4.2.1. Organic ligand exchange

Replacing oleate molecules with short ionic dithiol ligands such as 1,2-ethanedithiol (EDT) and 1,3-benzenedithiol (BDT) is a promising strategy to improve connectivity in PbS CQD films [106–111]. Some groups, however, have reported poor resistance to ambient atmospheric conditions [112,113]. Later on, optimized ligand exchanged employing 3-mercaptopropionic acid (MPA) seemed to lead to better stability, improved mobility, and the resulting film was less influenced by possible trap states for similar interparticle spacing (Figure 13(c) and 13(d)) [114]. Jeong et al*.* [115] suggested that the greater chemical diversity of MPA (thiol + carboxylic groups) in comparison to EDT can be responsible for passivating a broader distribution of surface states. Through modelling and X-ray photoelectron spectroscopy analysis, it was found however that the ligand coverage efficiency, and thus the stability of the particle surface, could be hindered by hydroxylation mechanisms [116,117]. It was also reported that substituting the oleate group for oleylamine [118] or octylamine [119] through a three-day solution-phase ligand exchange prior to the MPA or EDT solid phase exchange promoted a more effective replacement and improved passivation. More recently, Giansante et al*.* [120] reported a complete study of PbS CQDs passivated with various types of short conjugated ligands and were able to enhance their broadband light absorption while maintaining their stability.

#### 4.2.2. Inorganic ligand exchange

**Figure 14.**
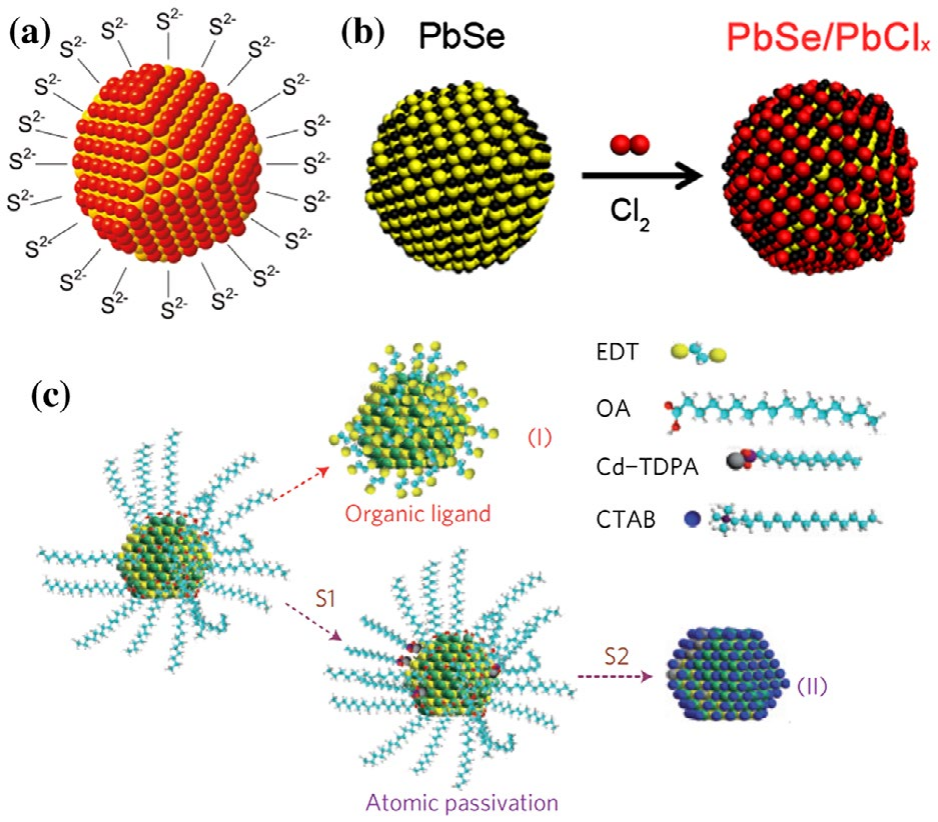
(a) S^2–^ inorganic capping. Reprinted with permission from [123]. Copyright 2011 American Chemical Society. (b) Atomic-chlorine passivation. Reprinted with permission from [126]. Copyright 2012 American Chemical Society. (c) Atomic-ligand passivation developed by Tang et al*.* OA stands for oleic acid, TDPA for tetradecylphosphonic acid and CTAB for cetyltrimethylammonium bromide. Reprinted with permission from [96]. Copyright 2011 Nature Publishing Group.

Because of their bulkiness as well as their vulnerability to thermal degradation and oxidation, other researchers have aimed to substitute organic ligands for their inorganic analogues. Talapin’s group started by using Sn_2_S_6_^4–^ ions to cap various types of quantum dots (CdSe, CdTe, CdS, Bi_2_S_3_, Au, Pd) and extended this work further with a wider range of inorganic ligands such as HS^–^, Se^2–^, HSe^–^, Te^2–^, HTe^–^, TeS_3_^2–^, OH^–^, NH^2–^ and S^2–^ (Figure 14(a)) [121–123]. In a similar vein, Yang et al*.* [124] tuned the external quantum efficiency by supressing Auger recombination through adjusting the composition of the outer and intermediate shells of core-shells structure. Supran et al*.* [125] also improved shortwave-infrared device performance by engineering PbS-CdS core-shell CQD in a type IV LED (organic-CQD-inorganic structure). Other promising methods include atomic chlorine ligand passivation (Figure 14(b)), leading to improved electronic transport [126,127]. After completely removing the oleate ligand using ammonium sulphide, Zhang et al*.* [128] reported that the remaining QDs were self-passivated and interconnected through metal-sulfide bonding. Cate et al*.* [129] observed the activation of carrier multiplication after infilling PbSe films with Al_2_O_3_ or Al_2_O_3_/ZnO by atomic layer deposition using microwave conductivity transients. Kinder et al*.* [130] assembled various solar cells that could reach an efficiency of 2.4% from a matrix of PbS QDs encapsulated in a CdS matrix, creating a quasi-superlattice[Bibr CIT0130].

In their inorganic halide ligand passivation method, Tang et al*.* [96] successfully improved passivation due to surface sulfur dangling bonds by treating the quantum dots in a mixture of tetradecylphosphonic acid, CdCl_2_, and oleylamine (60°C, 5 min) (Figure 14(c)). This improved the stability and size distribution by removing certain surface defects. The spin-coating process took place in a glove box where each sub-layer was then post-treated with solutions of cetyltrimethylammonium bromide (Br^–^), hexadecyltrimethylammonium chloride (Cl^–^), or tetrabutylammonium iodide (I^–^). These treatments improved the device performance significantly [131,132]. Thon et al*.* [133] also studied the evolution of mid-gap trap-states after such a passivation by *ab initio* calculations. Ip et al*.* [97] performed further optimization, leading to a solar cell with 7.4% efficiency.

#### 4.2.3. Transport in CQD depleted heterojunction solar cells

**Figure 15.**
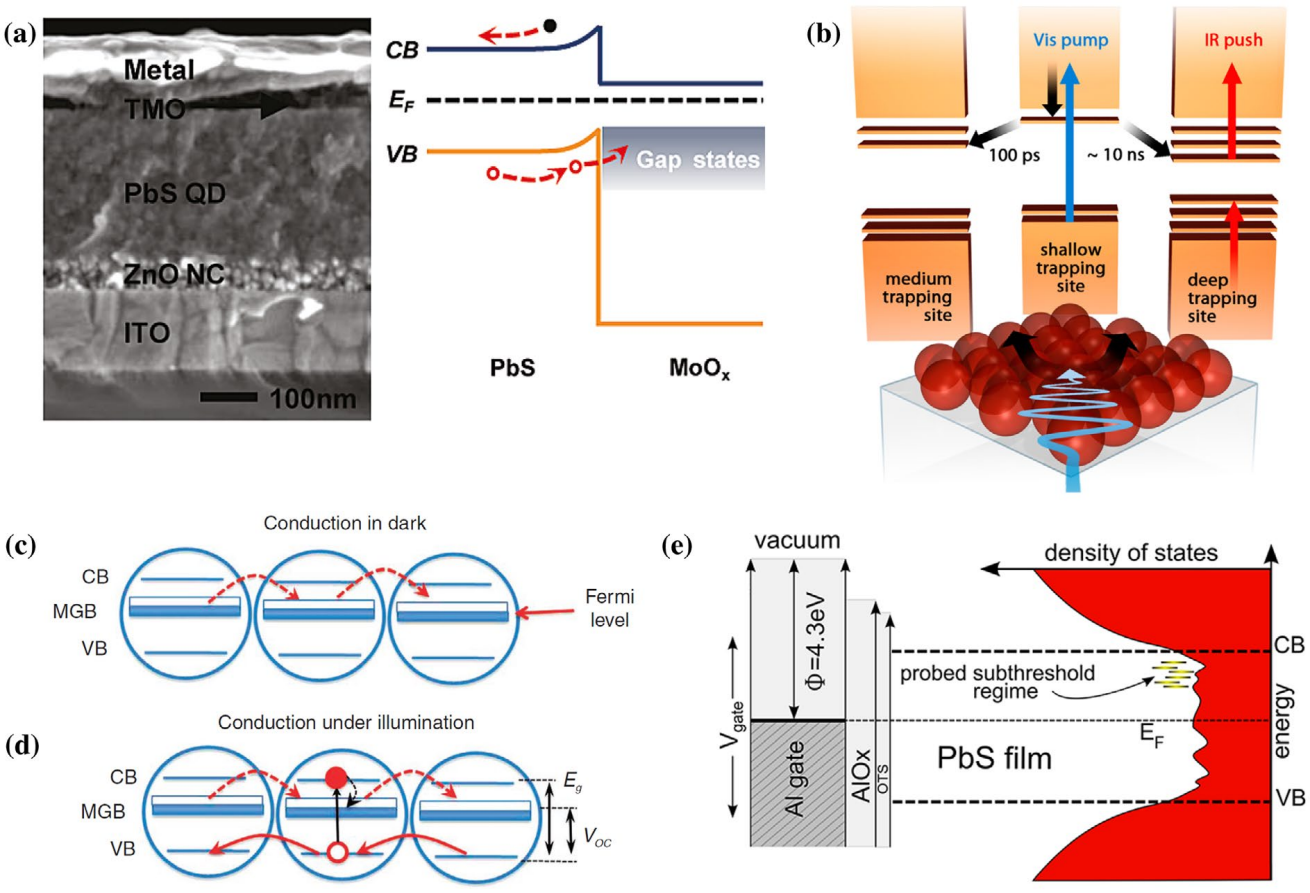
**(**a) Cross-sectional scanning electron microscopy image and band structure of a device with MoO_X_ selective contact. TMO stands for transition metal oxide, NC for nanocrystal, ITO for indium tin oxide, CB for conduction band and VB for valence band. Reprinted with permission from [147]. Copyright 2011 American Chemical Society. (b) Push pump photocurrent method to probe trap states. Reprinted with permission from [149]. Copyright 2013 American Chemical Society. Mid-gap band (MGB) conduction (c) in the dark and (d) under illumination along with simulated density of states. Reprinted with permission from [153]. Copyright 2011 Nature Publishing Group. (e) Band diagram showing a schematic density of states in the quantum dot film on the right. Reprinted with permission from [154]. Copyright 2013 American Chemical Society.

Recently, the term *selective contact* has been considered as a better description for the role of these collecting junctions, compared to *heterojunctions*. Mora-Sero et al*.* [134] clearly observed how the choice of material can literally screen the charges: fluorine doped tin oxide, Au or poly(3,4-ethylenedioxythiophene) (PEDOT) for holes; TiO_2_ or ZnO for electrons. It was also reported that major back surface recombination mechanisms can be suppressed by simply adding hole [135] or electron [136] selective contacts. The appropriate selection of materials and doping techniques have been at the centre of considerable attention when it comes to engineering the interface between PbS CQDs and the electron selective contact [137–141]. Hole collection was successfully improved by using LiF in Schottky devices [142–145], while DHJSC shows better results using MoO_X_ (see Figure 15(a)) [146,147]. Gao et al*.* [148] also reported that hole injection could be controlled through Schottky barrier engineering. This could be achieved by align the work function of the metal with the energy bands of the PbS CQDs with specific sizes.

Bakulin et al*.* [149] observed that charge immobilization and poor charge separation were caused by the presence of trap states with various depths (0.3–0.5 eV) below the conduction band (see Figure 15(b)). Using 1D and 3D models and taking into consideration the geometry of the device and its photoluminescence response, Zhitomirsky [150] calculated the lifetime, trap density, mobility, and diffusion coefficient. He found that state-of-the-art devices have an effective diffusion length of 80 nm. Recently, Whitham et al*.* [151] demonstrated that charge localization can be greatly suppressed by reducing the level of disorder in CQD films, by epitaxially connecting ordered PbSe nanocrystals.

Surface passivation can have a considerable impact on transport in CQD films which is substantially mediated by intraband (in-gap) states [152]. Using an optical field-effect transistor configuration, Nagpal and Klimov [153] described the existence of a *mid*-*gap band* with different levels of participation to charge transport, depending on whether the device is in the dark or under illumination (Figure 15(c–e)). Using similar methods, Stadler et al*.* [154] employed sub-threshold analysis to determine the trap distribution and map the density of state distribution in CQD films.

## 5. Concluding remarks and outlook

A tremendous effort has been deployed to analyse and exploit the properties of nanostructures such as quantum dots in order to assess their applicability in the field of photovoltaic and other optoelectronic devices. While theoretical speculations and calculations place these materials at the centre of the third-generation solar cells, recent research output tends to demonstrate that many unpredictable issues arise from the implementation of such structures inside devices. The considerable work targeting material synthesis and device engineering, however, is gradually circumventing these hindrances, opening the door to a potential solar cell technology which could be entirely fabricated through chemical processes and thus, at lower costs. For example, recent attempts have been aiming to hybridize PbS quantum dots with methylammonium lead halide perovskite, and achieved an unprecedented efficiency of 10.6% [155–157]. Nowadays, extensive research aims at nanostructuring a wide range of materials [158–160], including the promising lead halide perovskite [161–164], to further improve the efficiencies and versatility of nanocrystal-based optoelectronic devices.

## Disclosure statement

No potential conflict of interest was reported by the authors.
